# Incidence and Prevalence of Peripheral Arterial Disease in South Korea: Retrospective Analysis of National Claims Data

**DOI:** 10.2196/34908

**Published:** 2022-11-18

**Authors:** Gi Wook Ryu, Young Shin Park, Jeewuan Kim, Yong Sook Yang, Young-Guk Ko, Mona Choi

**Affiliations:** 1 Department of Nursing Hansei University Gunpo-si Republic of Korea; 2 Mo-Im Kim Nursing Research Institute College of Nursing Yonsei University Seoul Republic of Korea; 3 College of Nursing Yonsei University Seoul Republic of Korea; 4 Department of Statistics and Data Science Yonsei University Seoul Republic of Korea; 5 Division of Cardiology Severance Cardiovascular Hospital College of Medicine, Yonsei University Seoul Republic of Korea

**Keywords:** peripheral arterial disease, insurance claims, incidence, prevalence, endovascular revascularization, amputation, population-based study, blood flow, intermittent claudication, age, sex

## Abstract

**Background:**

Peripheral arterial disease (PAD) causes blood vessel narrowing that decreases blood flow to the lower extremities, with symptoms such as leg pain, discomfort, and intermittent claudication. PAD increases risks for amputation, poor health-related quality of life, and mortality. It is estimated that more than 200 million people worldwide have PAD, although the paucity of PAD research in the East detracts from knowledge on global PAD epidemiology. There are few national data–based analyses or health care utilization investigations. Thus, a national data analysis of PAD incidence and prevalence would provide baseline data to enable health promotion strategies for patients with PAD.

**Objective:**

This study aims to identify South Korean trends in the incidence and prevalence of PAD and PAD treatment, in-hospital deaths, and health care utilization.

**Methods:**

This was a retrospective analysis of South Korean national claims data from 2009 to 2018. The incidence of PAD was determined by setting the years 2010 and 2011 as a washout period to exclude previously diagnosed patients with PAD. The study included adults aged ≥20 and <90 years who received a primary diagnosis of PAD between 2011 and 2018; patients were stratified according to age, sex, and insurance status for the incidence and prevalence analyses. Descriptive statistics were used to assess incidence, prevalence, endovascular revascularization (EVR) events, amputations, in-hospital deaths, and the health care utilization characteristics of patients with PAD.

**Results:**

Based on data from 2011 to 2018, there were an average of 124,682 and 993,048 incident and prevalent PAD cases, respectively, in 2018. PAD incidence (per 1000 persons) ranged from 2.68 to 3.09 during the study period. From 2012 to 2018, the incidence rate in both sexes showed an increasing trend. PAD incidence continued to increase with age. PAD prevalence (per 1000 persons) increased steadily, from 3.93 in 2011 to 23.55 in 2018. The number of EVR events varied between 933 and 1422 during the study period, and both major and minor amputations showed a decreasing trend. Health care utilization characteristics showed that women visited clinics more frequently than men, whereas men used tertiary and general hospitals more often than women.

**Conclusions:**

The number of incident and prevalent PAD cases generally showed an increasing trend. Visits to tertiary and general hospitals were higher among men than women. These results indicate the need for attention not only to Western and male patients, but also to Eastern and female patients with PAD. The results are generalizable, as they are based on national claims data from the entire South Korean population, and they can promote preventive care and management strategies for patients with PAD in clinical and public health settings.

## Introduction

Peripheral arterial disease (PAD) is a major vascular condition that decreases blood flow to the affected limbs; it is mostly caused by atherosclerosis, a progressive disease characterized by the intra-arterial accumulation of lipids and fibrous elements [1,2]. The PAD symptoms of claudication and critical limb ischemia (CLI) occur following the reduction of blood flow to affected limbs, with resultant resting pain and cramps [3-5]. Worldwide, more than 236 million people are affected by PAD, and the PAD burden could increase with population aging [6,7], as PAD prevalence consistently and globally increases with age, especially in older age groups [6,7]. In the United States, treatment of CLI symptoms in older patients (aged >65 years) with PAD incurs an estimated cost of US $1.2 billion yearly [8,9].

Vessel patency in the affected limb is essential for adequate blood flow, as vessel obstruction increases risk for amputation, mortality, and poor health-related quality of life [10-12]. Endovascular revascularization (EVR) by percutaneous transluminal angiography (PTA) is the preferred method to open affected vessels, thereby improving the clinical manifestations of claudication and CLI [13] and reducing major amputations of the affected limbs [14]. However, the prognosis of the surgical procedure is associated with procedural characteristics, such as method and target region, and patient characteristics, such as age, smoking, and comorbidities [15]. A systematic review revealed that major amputation events after surgical intervention were significantly related to comorbidities, such as cardiovascular disease, chronic kidney disease, diabetes, chronic occlusive pulmonary disease, dementia, and frailty [16].

Some patients with PAD have atypical presentation, without intermittent claudication or clear limb symptoms [4,9], and may attempt to alleviate limb symptoms by reducing physical activity, which may eventually cause a worse prognosis [4,17]. Thus, patients with asymptomatic PAD may not be properly diagnosed and may not receive adequate treatment [18,19]. Furthermore, chronic diseases such as PAD affect psychological well-being by inducing depression, anxiety, and low quality of life [20,21]. Pain and difficulty in walking distances and climbing stairs in patients with PAD are significantly related to quality of life [22] and well-being [23].

Understanding trends in the incidence, prevalence, and clinical manifestations of PAD and related procedures, treatments, and health outcomes are crucial for public health interventions. Thus, identifying the incidence and prevalence of PAD using recent national data may provide baseline data to facilitate the development of health promotion strategies and interventions for patients with PAD and public health promotion. However, most previous studies have examined the incidence and prevalence in Western countries [1,24], and PAD has been studied only as part of atherosclerotic disease [25].

Currently, studies on PAD in Eastern countries are scarce, which limits understanding of the global features of PAD. Moreover, few studies have investigated national data on health care utilization characteristics. This study used nationwide data obtained from the Health Insurance Review and Assessment (HIRA) Service of South Korea from 2011 to 2018 to investigate (1) trends in the incidence, prevalence, and treatment of PAD (eg, EVR events) and PAD-related amputations and in-hospital deaths and (2) health care utilization characteristics of patients with PAD.

## Methods

### Ethical Considerations

This study was reviewed by the Yonsei University health system institutional review board (Y-2019-0105) and was conducted using secondary data analysis with a descriptive study design. This study used South Korea–specific research data obtained by HIRA (M20190923977).

### Data Source

We acquired data from the HIRA database for patients with PAD from January 1, 2009, to December 31, 2018. The National Health Insurance system in Korea is a single-payer system that covers 98% of the total population. More than 99% of medical institutions are mandatorily included in the system, and the HIRA collects claims data to reimburse health care providers [26]; these data cover all South Korean citizens and can be used as anonymized information on diagnoses, procedures, prescription records, demographic information, and direct medical costs [26,27].

### Study Population

Patients with PAD were defined as those with the following Korean Standard Classification of Diseases, 7th revision (KCD-7) codes: I70.2, I73.9, I73.9, I74.3, I74.4, I74.5, I74.8, and I74.9; these are primary PAD diagnoses (Multimedia Appendix 1). The KCD-7 codes were developed in Korea based on the International Classification of Diseases, Tenth Revision (ICD-10) codes, and the KCD-7 codes for PAD are identical to the ICD-10 codes. We selected the codes by referring to published studies that analyzed PAD-related data with similar codes [24,25,28]. Adolescents (ie, those aged 19 years or younger) were excluded. Adult patients (aged ≥20 and <90 years) who were diagnosed with PAD by a physician between January 1, 2009, and December 31, 2018, as outpatients or inpatients at health facilities ranging from clinics to tertiary hospitals were enrolled.

### Trends in Incidence and Prevalence

**Figure 1:**
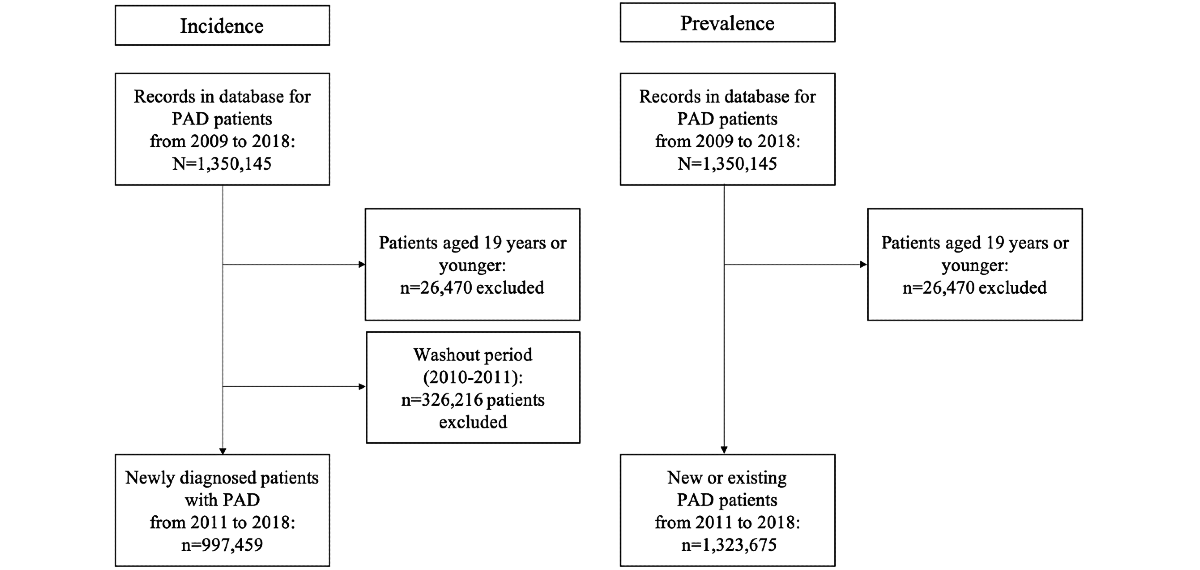
Selection of patients with peripheral arterial disease using Health Insurance Review and Assessment data. PAD: peripheral arterial disease.

For PAD incidence, data from 2009 to 2018 were used. Data from patients treated for PAD from January 1, 2009, to December 31, 2010, were excluded to identify newly diagnosed patients. In general, the incidence of chronic diseases, such as diabetes and PAD, is calculated after excluding data from a 2-year washout period [14], and the same method was used in this study. The index date was defined as the date between 2011 and 2018 on which a patient was first diagnosed with PAD, and these dates were analyzed to determine PAD incidence. PAD prevalence was ascertained from data from 2011 to 2018 to identify patients treated for PAD every year. The index date was determined as the date on which a patient was first diagnosed for every year from 2011 to 2018 (Figure 1).

The annual number of incident and prevalent PAD cases and PAD incidence and cumulative prevalence were assessed. PAD incidence and prevalence are reported as the number of patients with PAD per 1000 individuals.

### Trends in Treatment and Deaths

To identify the annual number of EVR events, open surgical procedures, amputations, and in-hospital deaths, the numbers of cases from January 1, 2011, to December 31, 2018, were assessed based on the prevalence database used in this study. The codes for EVR events, surgical procedures, and amputations were selected based on a previous study [25] (Multimedia Appendix 2). EVR events included PTA, stent grafts, and atherectomies, whereas amputations included major and minor amputations. In-hospital death was assessed based on the results of medical treatment for patients diagnosed with PAD from January 1, 2011, to December 31, 2018.

### Health Care Use Characteristics

To identify health care use characteristics, we assessed all claims from January 1, 2011, to December 31, 2018, and grouped them to identify the number of visits to tertiary hospitals, general hospitals, small hospitals, long-term care facilities, and clinics. Tertiary hospitals are defined as large hospitals with more than 20 medical departments, with each department having relevant specialists. General hospitals are defined as having 100 or more beds, and hospitals are defined as small hospitals with 30 or more beds. Long-term care facilities provide medical and nursing care for inpatients and outpatients. Clinics provide treatment and care to outpatients.

### Statistical Analysis

The number of incident PAD cases was the number of patients who were newly diagnosed with PAD. The annual PAD incidence was calculated as the number of newly diagnosed patients with PAD in a year divided by the size of the population at risk. The “population at risk” for this calculation was defined by excluding preexisting patients with PAD from the midyear population [29,30]. As we had already excluded patients from 2010 from the analysis, the incidence could not be calculated for 2011, and this study therefore only analyzed incidence from 2012 to 2018.

The number of prevalent cases was the number of patients who were previously or newly diagnosed with PAD and underwent treatment, whereas PAD prevalence was the total accumulated number of patients with PAD every year. In this study, census data were used for the total population of South Korea.

Incident cases, incidence, prevalent cases, prevalence, EVR events, open surgical procedures, amputations, in-hospital deaths, and health care utilization characteristics were analyzed with descriptive statistics. The frequency of PAD incident cases was stratified according to age, sex, and insurance status. Incidence was adjusted by age and sex using a standardization method that calculates a weight based the study population for the year 2011 [31]. The incidence trends were analyzed according to sex and age.

Changes in the frequency of EVR events, open surgical procedures, and amputations per year were assessed to determine treatment trends. For the analysis of health care utilization, all claims were grouped by sex to identify the number of visits to tertiary hospitals, general hospitals, small hospitals, long-term care facilities, and clinics.

SAS (version 9.3; SAS Institute, Inc) and R (2020 version, R Foundation for Statistical Computing) were used for statistical analysis.

## Results

### Demographics

A total of 997,459 new patients with PAD from 2011 to 2018 were identified. In 2011 and 2018, the numbers of new patients with PAD were 117,876 and 142,211, respectively. The total number of new female patients was 603,788 (60.5%), which was greater than the number of new male patients (n=393,671, 39.5%). Among patients who were newly diagnosed with PAD during the study period, those in their 50s were the most common by age at 242,425 (24.3%). In 2018, the number of prevalent PAD cases was 993,048. From 2011 to 2018, the number of prevalent PAD cases consistently increased (Table 1).

**Table 1:** Incident and prevalent cases of peripheral arterial disease from 2011 to 2018 in South Korea. Data represent the number of patients with peripheral arterial disease. Percentages are based on incident cases of peripheral arterial disease.

				2011		2012		2013		2014		2015		2016		2017		2018		Total (2011-2018)		Average
Incident cases, n				117,876		102,153		111,345		116,025		125,674		139,191		142,984		142,211		997,459		124,682
**Incident cases by sex** **, n (%)**																						
		Male	46,399 (39.4)		41,601 (40.7)		44,484 (40)		45,503 (39.2)		49,185 (39.1)		53,495 (38.4)		55,981 (39.2)		57,023 (40.1)		393,671 (39.5)		49,209	
		Female	71,477 (60.6)		60,552 (59.3)		66,861 (60)		70,522 (60.8)		76,489 (60.9)		85,696 (61.6)		87,003 (60.9)		85,188 (59.9)		603,788 (60.5)		75,474	
**Incident cases by age group (years),** **n (%)**																						
	20s			3527 (3.0)		2967 (2.9)		3090 (2.7)		3044 (2.7)		3415 (2.7)		3863 (2.8)		3853 (2.7)		4086 (2.9)		27,845 (2.8)		3481
	30s			7451 (6.3)		6125 (6.0)		6375 (5.7)		6172 (5.3)		6464 (5.1)		7215 (5.2)		7249 (5.1)		7100 (5.0)		54,151 (5.4)		6769
	40s			17,343 (14.7)		14,295 (14.0)		14,982 (13.5)		15,087 (13.0)		15,896 (12.6)		17,069 (12.3)		17,101 (12.0)		16,177 (11.4)		127,950 (12.8)		15,994
	50s			29,491 (25.0)		25,948 (25.4)		27,616 (24.8)		28,696 (24.7)		30,470 (24.2)		33,346 (24.0)		33,748 (23.6)		33,110 (23.3)		242,425 (24.3)		30,303
	60s			27,907 (23.7)		23,870 (23.4)		25,765 (23.1)		26,873 (23.2)		29,617 (23.6)		34,356 (24.7)		35,952 (25.1)		36,541 (25.7)		240,881 (24.1)		30,110
	70s			24,647 (20.9)		22,185 (21.7)		25,617 (23.0)		26,834 (23.1)		29,117 (23.2)		31,344 (22.5)		31,977 (22.4)		31,759 (22.3)		223,480 (22.4)		27,935
	80s			7510 (6.4)		6763 (6.6)		7946 (7.1)		9273 (8.0)		10,695 (8.5)		11,998 (8.6)		13,104 (9.2)		13,438 (9.4)		80,727 (8.1)		10,091
**Incident cases by insurance** **status, n (%)**																						
		Health insurance	108,481 (92.0)		94,597 (92.6)		103,333 (92.8)		107,898 (93.0)		116,467 (92.7)		128,866 (92.6)		132,635 (92.8)		132,165 (92.9)		924,442 (92.7)		115,555	
		Medical aid	9250 (7.8)		7458 (7.3)		7911 (7.1)		8032 (6.9)		9133 (7.3)		10,248 (7.4)		10,219 (7.1)		9984 (7.0)		72,235 (7.2)		9029	
		Veteran	145 (0.1)		98 (0.1)		101 (0.1)		95 (0.1)		74 (0.1)		77 (0.1)		130 (0.1)		62 (0.0)		782 (0.1)		98	
Prevalent cases^a^, n				154,296		256,449		367,794		474,627		595,621		725,163		854,630		993,048		993,048		N/A^b^

^a^Prevalent cases refers to patients who were undergoing treatment after diagnosis of peripheral arterial disease; the values are accumulated values.

^b^N/A: not applicable.

### Overall Trends in Incidence and Prevalence

In 2012, the total PAD incidence per 1000 patients was 2.92, and the absolute change was 0.07 between 2012 and 2018. The trend did not noticeably increase or decrease (Table 2).

**Figure 2:**
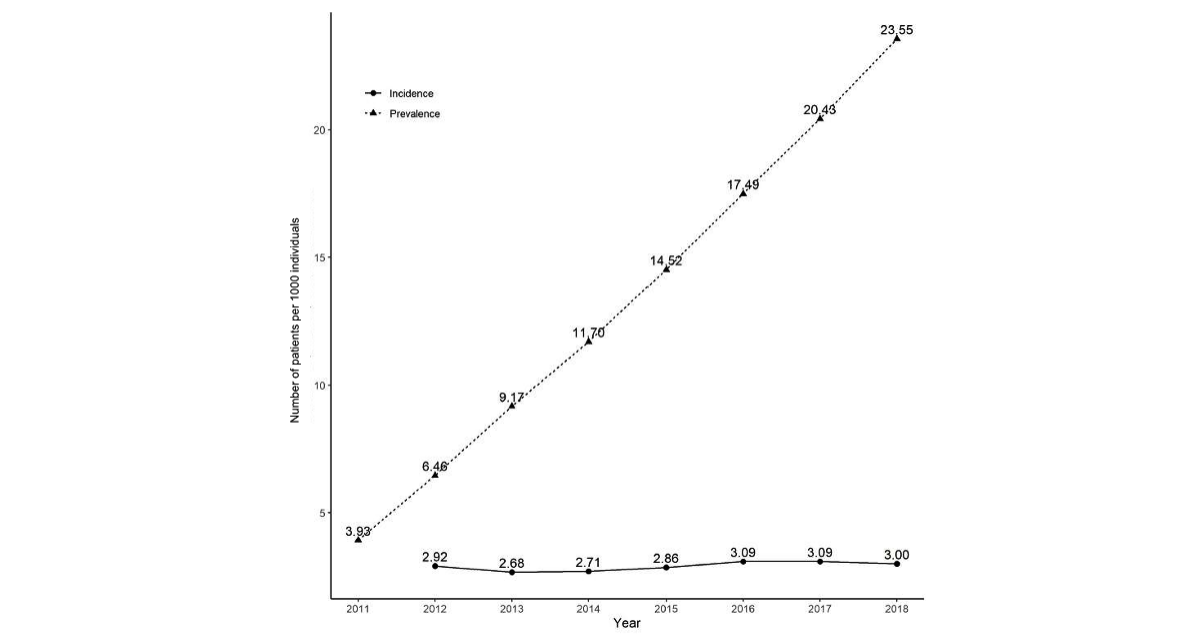
Trends in incidence and prevalence. Incidence was adjusted by age and sex.

The total incidence trend per year showed an increase from 2012 to 2018 (Figure 2).

**Figure 3:**
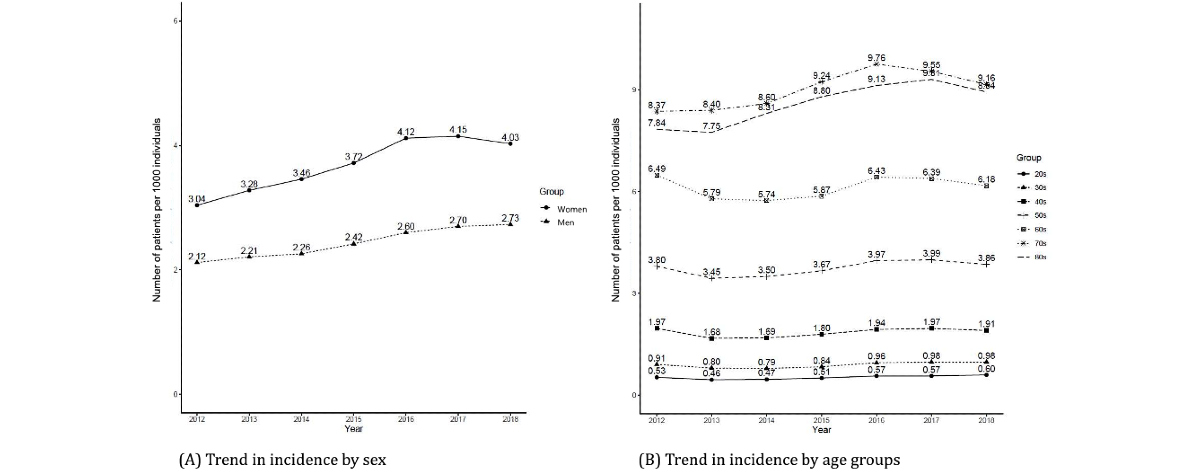
Trends in incidence by sex and age groups.

The incidence in men increased from 2.12 per 1000 individuals in 2012 to 2.73 per 1000 individuals in 2018, for an absolute increase of 0.61. In women, the incidences in 2012 and 2018 were 3.04 and 4.03 per 1000 individuals, respectively, for an absolute increase of 0.99. From 2012 to 2018, the incidence trend was consistently higher in women than men (Figure 3A).

PAD incidence continued to increase with age from 20 to 70 years, and the average incidence among those in their 80s or older was higher than among those in their 70s. Among individuals in their 80s, PAD incidence in 2012 and 2018 was 7.84 and 8.94, respectively, for an absolute increase of 1.10, which was the highest among all age groups. In 2012 and 2018, the prevalence was 6.46 and 23.55, respectively, representing a consistently increasing trend. As shown in Figure 3B, the slope showed an increasing trend without a plateau.

**Table 2:** Incidence and prevalence per 1000 individuals. Peripheral arterial disease incidence and prevalence are based on the number of incident cases and the overall South Korean population. Incidence was adjusted by age and sex.

Year		2011	2012	2013	2014	2015	2016	2017	2018	AC^a^ (2012-2018)	Average (2012-2018)
Incidence		N/A^b^	2.92	2.68	2.71	2.86	3.09	3.09	3.00	+0.07	2.91
**Incidence by sex**											
	Male	N/A	2.12	2.21	2.26	2.42	2.60	2.70	2.73	+0.61	2.43
	Female	N/A	3.04	3.28	3.46	3.72	4.12	4.15	4.03	+0.99	3.69
**Incidence by age (years)**											
	20s	N/A	0.53	0.46	0.47	0.51	0.57	0.57	0.60	+0.07	0.53
	30s	N/A	0.91	0.80	0.79	0.84	0.96	0.98	0.98	+0.07	0.89
	40s	N/A	1.97	1.68	1.69	1.80	1.94	1.97	1.91	–0.06	1.85
	50s	N/A	3.80	3.45	3.50	3.67	3.97	3.99	3.86	+0.06	3.75
	60s	N/A	6.49	5.79	5.74	5.87	6.43	6.39	6.18	–0.31	6.13
	70s	N/A	8.37	8.40	8.60	9.24	9.76	9.55	9.16	+0.79	9.01
	80s	N/A	7.84	7.75	8.31	8.80	9.13	9.31	8.94	+1.10	8.58
Prevalence		3.93	6.46	9.17	11.70	14.52	17.49	20.43	23.55	+19.62	13.41^c^

^a^AC: absolute change.

^b^N/A: not applicable.

^c^Average prevalence is the average from 2011 to 2018.

### Trends in Treatment and Death

**Figure 4:**
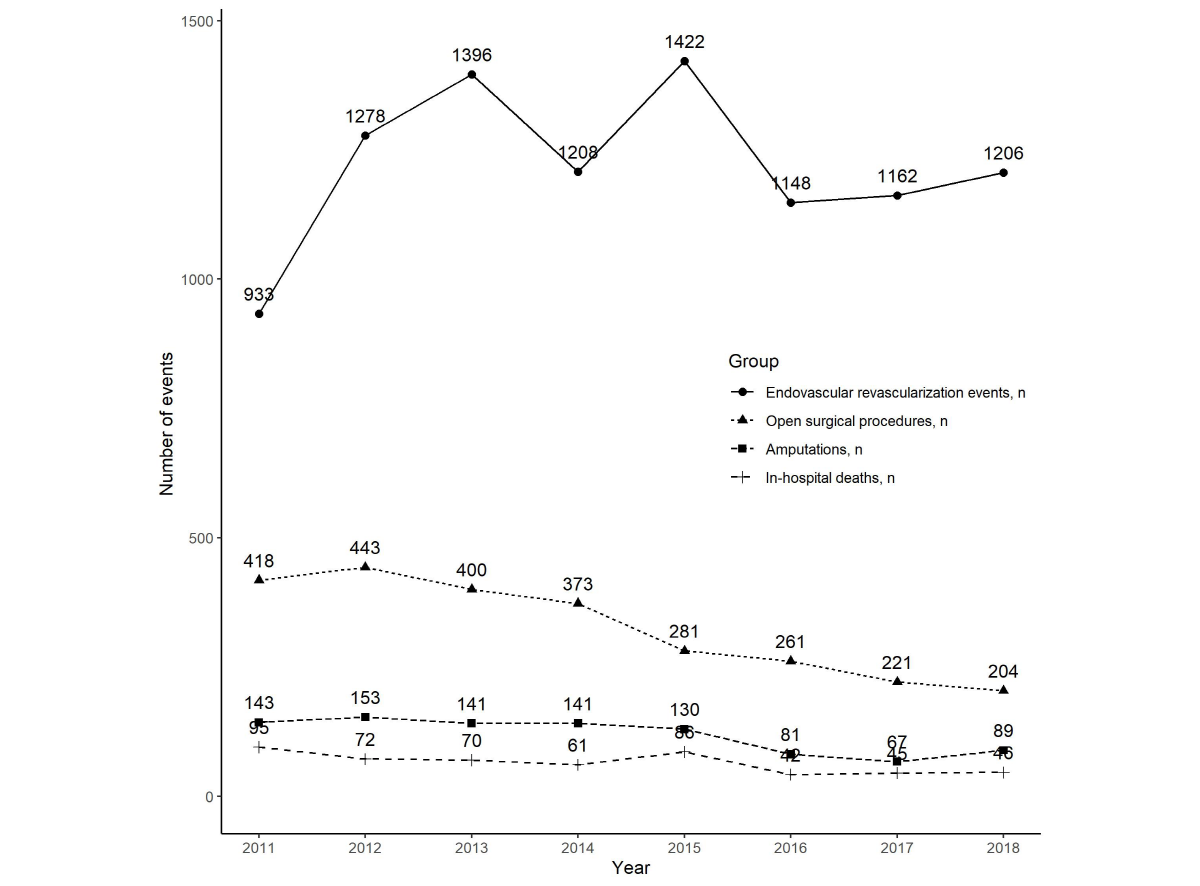
Trends in the annual numbers of endovascular revascularization events, open surgical procedures, amputations, and all-cause in-hospital deaths.

From 2011 to 2018, the number of EVR events showed fluctuations (Figure 4).

In 2011, 933 EVR events were observed, increasing to 1206 cases in 2018, an absolute increase of 273 cases. In the same period, amputations decreased from 143 to 89, an absolute decrease of 54 cases. Major amputations decreased from 61 in 2011 to 35 in 2018, and minor amputations decreased from 82 in 2011 to 54 in 2018.

In-hospital deaths decreased from 95 in 2011 to 46 in 2018, an absolute decrease of 49. The number of in-hospital deaths was greater within 7 days than between 30 and 90 days. In 2011, 53 and 89 in-hospital deaths occurred within 7 and 30 days, respectively. In-hospital deaths within 30 days included deaths within 0 days, and in 2011, there were 36 deaths between 7 and 30 days. In 2018, 33 and 44 in-hospital deaths were observed within 7 days and 30 days, respectively (Table 3).

**Table 3:** Annual number of endovascular revascularization events, open surgical procedures, amputations, and all-cause in-hospital deaths.

			2011, n		2012, n		2013, n		2014, n		2015, n		2016, n		2017, n		2018, n		Total (2011-2018), n		AC^a^ (2011-2018)		Average (2011-2018), n
EVR^b^ events			933		1278		1396		1208		1422		1148		1162		1206		9753		+273		1219
Open surgical procedures			418		443		400		373		281		261		221		204		2601		–214		325
**Amputations**																							
	Total	143		153		141		141		130		81		67		89		945		–54		118	
	Major amputations	61		68		59		56		47		28		27		35		381		–26		48	
	Minor amputations	82		85		82		85		83		53		40		54		564		–28		71	
**In-hospital deaths**																							
	Total	95		72		70		61		86		42		45		46		517		–49		65	
	Within 7 days	53		37		37		47		48		24		33		33		312		–20		39	
	Within 30 days	89		64		64		58		82		41		42		44		484		–45		61	
	Within 90 days	95		72		70		61		86		42		45		46		517		–49		57	

^a^AC: absolute change.

^b^EVR: endovascular revascularization.

### Health Care Utilization by Sex

Total claims from 2011 to 2018 were 4,222,726. Male patients used tertiary hospitals more than than female patients (n=177,274, 9.8% vs n=76,636, 3.2%, respectively). Female patients used clinics more than male patients (n=2,027,490, 83.8% vs n=1,222,519, 67.8%, respectively) (Table 4).

**Table 4:** Types of health care utilization based on claims by sex (N=4,222,726). Tertiary hospitals are large, with at least 20 medical departments and specialists for each department. General hospitals have at least 100 beds and hospitals at least 30. Long-term care facilities treat inpatients and outpatients. Clinics treat outpatients.

	Men			Women		
	Total (n=1,802,405), n (%)	Inpatients (n=30,402), n (%)	Outpatients (n=1,772,003), n (%)	Total (n=2,420,321), n (%)	Inpatients (n=11,198), n (%)	Outpatients (n=2,409,123), n (%)
Tertiary hospitals	177,274 (9.8)	14,173 (46.6)	163,101 (9.2)	76,636 (3.2)	3904 (34.9)	72,732 (3)
General hospitals	307,692 (17.1)	12,778 (42)	294,914 (16.6)	196,391 (8.1)	4118 (36.8)	192,273 (8)
Small hospitals	85,367 (4.7)	1922 (2.3)	83,445 (4.71)	106,292 (4.4)	1621 (14.5)	104,671 (4.3)
Long-term care facilities	9553 (0.5)	1294 (4.3)	8259 (0.47)	13,512 (0.6)	1226 (11)	12,286 (0.5)
Clinics	1,222,519 (67.8)	235 (0.8)	1,222,284 (69)	2,027,490 (83.8)	329 (2.9)	2,027,161 (84.2)

## Discussion

This study identified the incidence and prevalence of PAD and PAD treatment trends, in-hospital deaths, and health care utilization in South Korea over the past 8 years through a retrospective analysis of national claims data.

The average PAD incidence was 2.91 per 1000 individuals from 2012 to 2018. Previously, a United States–based study used MarketScan data, which includes commercial, Medicare, and Medicaid health insurance data, to identify patients with a PAD or CLI diagnosis and found that the mean annual incidence of PAD was 2.34 [32]. A study conducted in the United Kingdom used a database of 11 million patients from 2000 to 2014 to search for symptomatic patients with PAD with at least 1 medical record in at least 2 years and found that the overall PAD incidence was 1.73 to 3.85 per 1000 individuals [33]. Our findings show that PAD incidence in South Korea was higher than in the United States and similar to the United Kingdom. Considering the characteristics of the participants, this study included claims with 1 PAD diagnosis in 8 years. However, if our analysis had used the same criteria as the study conducted in the United Kingdom, the PAD incidence in South Korea would have been lower.

The sex-stratified incidence and prevalence trends of PAD differed from those in previous studies and were higher in women than in men. The proportion of female patients with PAD ranged from 59.3% (60,552/102,153) to 61.6% (85,696/139,191), whereas for male patients, it ranged from 38.4% (53,495/139,191) to 40.7% (41,601/102,153). In a previous study, PAD incidence was 23.05 per 10,000 person-years in males, which was higher than the reported 12.37 per 10,000 person-years in females [33]. PAD has traditionally been reported to be a male-dominant disease [34]. However, PAD has recently been reported to affect women as much as men in the general population [34]. A systematic review reported that women had a slightly higher prevalence than men by the age of 75 years in high-income countries, measured by an arterial ankle brachial index (ABI) of 0.90 or less [6]. Classifying health care utilization by sex in this study revealed differences in claims between women and men. In terms of health care use, the number of tertiary hospital claims was high for men, whereas the number of clinic claims was high for women. In a Korean study, men accounted for a higher proportion than women of patients who received procedures at tertiary hospitals [35].

In our study, the PAD incidence trend among individuals in their 20s to 70s increased with age, which is similar to the findings of studies based in the United Kingdom [33] and United States [32]. Aging increases PAD-associated risk factors, such as hypertension, hyperlipidemia, and diabetes, and thereby increases the prevalence of PAD [2,36]. In terms of absolute change, patients with PAD in their 80s had the highest increase, at 1.10. Aging has increased the proportion of people in their 80s in the general population, and accordingly, the proportion of patients with PAD has also increased.

In our study, the prevalence of PAD was 3.93 and 23.55 per 1000 individuals in 2011 and 2018, respectively, indicating a steadily increasing trend, without decline. The prevalence of PAD has been reported to consistently increase [1,37]. In a study based on health insurance claims data in Germany, the number of prevalent cases of PAD consistently increased [24], which is similar to the findings of this study. In a meta-analysis, the prevalence of PAD was 5.56% in adults older than 25 years worldwide [6]. The results of this study are consistent with those of previous studies. Considering patients who do not visit hospitals due to having an asymptomatic condition, the incidence and prevalence of PAD may have been underestimated.

In this study, the number of EVR events fluctuated during the study period and increased from 933 in 2011 to 1206 in 2018. Similarly, an increasing trend in EVR events was reported for the US population from 1996 to 2011 among patients with PAD and diabetes [38]. PTA is recommended as the first-line revascularization intervention for PAD and is known to be effective, safe, and widely applicable, with few complications [13]; therefore, PTA has a positive effect on preventing major amputations [14], and the trend of PTA use has increased.

In our study, the rates of both major and minor amputations decreased slightly. The finding of a decreasing trend in major amputations over time is similar to the results of a study of patients with PAD based on health insurance claims data in Germany between 2008 and 2016 [24].

In this study, the numbers of patients who died in the hospital within 7 days and 30 days of a PAD diagnosis were 24 to 53 and 41 to 89, respectively, suggesting that a high proportion of in-hospital deaths occurred within 7 days. Patients with PAD may develop complications, such as ischemic myocardial and cerebrovascular events and sepsis [39-41], and older adults have a higher risk of complications than younger age groups [40]. Interventions and intensive monitoring of complications in the first month are necessary for hospitalized patients with PAD.

This study had some limitations. The data were claims data for health insurance that were collected through administrative processes and were not intended for research purposes. Furthermore, the previously entered diagnosis codes might not have been changed, or the doctor might have only entered the diagnosis and treatment codes that were required for the health insurance claims. Thus, the number of patients, procedures, or surgeries may have been underestimated. Furthermore, PAD was defined using disease codes only. Therefore, individuals who were not diagnosed with PAD but had CLI-associated symptoms and those with an ABI of less than 0.9 may have been excluded from the study, leading to underestimated results. As this was a retrospective study that was based on claims data, many potential confounders were not adjusted for in the analyses. Moreover, the use of descriptive statistics limits statistical inferences across study groups.

Therefore, it is necessary to carefully interpret our results on the incidence and prevalence of PAD; future studies that investigate this topic should adjust for confounders, such as risk factors, geographical heterogeneities, and medical disparities. Furthermore, we suggest that health outcomes, not only medical procedures and surgeries but also psychological well-being (eg, depression and anxiety) and quality of life, should be considered in association with PAD.

Our findings provide evidence for strategies for health promotion and intervention for patients with PAD and may help with strategies to manage risk factors, such as ceasing smoking, following a low-fat diet, and managing weight. The American Heart Association guidelines recommended walking as an exercise for controlling risk factors [15,42]. PAD causes pain when walking, which makes it difficult to carry out daily activities and can influence psychological health, such as by inducing depression and anxiety in patients with PAD. Therefore, the management of psychological health deserves attention in PAD care for aging populations.

In this study, increasing trends in incident cases and the prevalence of PAD in South Korea were observed between 2011 and 2018. PAD incidence was higher in women than men in this study. A strength of this study is that, methodologically, the epidemiological trends of the entire South Korean population and all patients with PAD in South Korea were ascertained through public data analysis. Furthermore, the health care utilization of patients with PAD was determined based on national data, which enables the generalization of results for the provision of information to undertake both prevention and treatment in the clinical setting and for further research.

## Appendix

Multimedia Appendix 1 Code for patients with peripheral arterial disease.

Multimedia Appendix 2 Procedure codes and names for patients with peripheral arterial disease.

## Abbreviations

ABI: arterial ankle brachial index
CLI: clinical limb ischemia
EVR: endovascular revascularization
HIRA: Health Insurance Review and Assessment
ICD-10: International Classification of Diseases, Tenth Revision
KCD-7: Korean Standard Classification of Diseases, 7th revision
PAD: peripheral arterial disease
PTA: percutaneous transluminal angiography
